# Preliminary Assessment of Mutagenicity and In Vivo Toxicity of Date Pit Ethanolic Extracts: Safety Screening for Circular Economy Applications

**DOI:** 10.3390/foods15122168

**Published:** 2026-06-16

**Authors:** Ana Rita Soares Mateus, João Vindeirinho, Khaoula Khwaldia, Joana Castro, Daniela Araújo, Angelina Pena, Matheus Lemos, Ana Rita Barata, Maria José Saavedra, Gonçalo Almeida, Ana Sanches Silva, Carina Almeida

**Affiliations:** 1 National Institute for Agricultural and Veterinary Research (INIAV), I.P., Rua dos Lagidos, Lugar da Madalena, Vairão, 4485-655 Vila do Conde, Portugal; anarita.mateus@iniav.pt (A.R.S.M.); jo.vindeirinho@gmail.com (J.V.); joana.castro@iniav.pt (J.C.); daniela.araujo@iniav.pt (D.A.); matheusguilhermelemos@gmail.com (M.L.); anarita.barata@iniav.pt (A.R.B.); goncalo.almeida@iniav.pt (G.A.); 2 University of Coimbra, Faculty of Pharmacy, Polo III, Azinhaga de Stª Comba, 3000-548 Coimbra, Portugal; apena@ff.uc.pt; 3 Associated Laboratory for Green Chemistry (LAQV) of the Network of Chemistry and Technology (REQUIMTE), Laboratory of Bromatology and Pharmacognosy, Faculty of Pharmacy, University of Coimbra, Polo III, Azinhaga de Stª Comba, 3000-548 Coimbra, Portugal; 4 Center for Study in Animal Science (CECA), Medical and Biomedical Sciences School, Fernando Pessoa University, Gondomar, 4420-096 Porto, Portugal; 5 Laboratory for Process Engineering, Environment, Biotechnology and Energy (LEPABE) Associate Laboratory in Chemical Engineering (ALiCE), Faculty of Engineering, University of Porto, 4200-465 Porto, Portugal; 6 Laboratory of Natural Substances (LSN), National Institute of Research and PhysicoChemical Analysis (INRAP), BiotechPole Sidi Thabet, Sidi Thabe, Ariana 2020, Tunisia; khaoulakhwaldia@gmail.com; 7 Centre of Biological Engineering, University of Minho, 4710-057 Braga, Portugal; 8 Centre for the Research and Technology of Agro-Environmental and Biological Sciences (CITAB), Institute for Innovation, Capacity Building and Sustainability of Agri-Food Production (Inov4Agro), University of Trás-os-Montes and Alto Douro, 5000-801 Vila Real, Portugal; saavedra@utad.pt; 9 Associate Laboratory for Animal and Veterinary Sciences (Al4AnimalS), 1300-477 Lisbon, Portugal

**Keywords:** date pits, date by-products, food safety, ames test, *Galleria mellonella*, toxicity

## Abstract

Date (*Phoenix dactylifera* L.) pits are a by-product of the date processing industry and are being explored as a source of bioactive compounds within the framework of the circular economy. This study aimed to perform a preliminary safety screening of the in vivo toxicity and mutagenic potential of ethanolic extracts obtained from different date pit varieties. Extracts from *Alig* (DA), *Deglet Nour* (DDN), and *Kentichi* (DK) varieties were administered to *Galleria mellonella* larvae at different concentrations, and health indices and survival were monitored for 4 days. No significant toxicity was observed at concentrations up to the MIC (6.25 mg/mL), while higher doses (≥2 MIC) had moderate effects on larval viability. The mutagenic potential was evaluated for two extracts (DDN and DK) using the Ames test with *Salmonella enterica* serovar *Typhimurium* strains TA98 and TA100, in the absence of metabolic activation. Mutagenic index values were below 2.0 for all conditions tested, with the exception of DDN on TA98, where values consistently exceeded 2.0 without a clear dose–response relationship. Due to the absence of metabolic activation and the limited strain panel, the results should be interpreted as preliminary. Overall, the combined preliminary in vitro and in vivo initial findings suggest that ethanolic extracts of date pits do not exhibit an evident mutagenic or toxic effects at biologically relevant concentrations under the conditions tested, providing a basis for further safety evaluation towards their application in the food industry.

## 1. Introduction

Dates are the fruits of date palm tree (*Phoenix dactylifera* L.), an economically and culturally important crop cultivated mainly in arid regions. Approximately 9.6 million tonnes were produced worldwide in 2023, with Egypt, Saudi Arabia and Iran as the main producers [1]. During industrial processing for pitted dates, date paste, or date syrup, large quantities of date pits or seeds are generated as a by-product, representing about 10–18% of the total fruit weight [2].

Traditionally, date pits have been used as animal feed, soil fertilizer, or as a source of date pit oil, which is rich in linolenic acid, for cosmetic and pharmaceutical applications [3]. Moreover, roasted date pits have been used as a caffeine-free coffee substitute, with a comparable aroma to coffee [4]. However, date pits contain significant levels of phenolic compounds, including phenolic acids (gallic, ferulic, caffeic, *p*-coumaric, protocatechuic, and vanillic acids) and flavonoids (rutin, catechin, quercetin, luteolin, and kaempferol) [2,3,4,5]. These bioactive components are associated with antioxidant and antimicrobial properties. In our previous comparative analysis of fruit seeds (including date, grape and cherry), we showed that date pits exhibit notably higher antioxidant capacity than the other by-products [5].

Following the principles of the circular economy, numerous studies have explored new applications for date pits, since they are also abundant in vitamins, fatty acids, amino acids, dietary fiber, and other healthy ingredients. For example, date pit powder has been incorporated into food formulations as a functional ingredient, such as a partial flour substitute in pita bread [6], as a natural sweetener or fiber source in bakery products [7], and as a natural preservative in meat products [8]. Moreover, date pit extracts have been used as active components in food packaging due to their antioxidant capacity [9] and as a source of natural bioactive extracts, such as gallic acid [10].

Beyond food applications, date pit extracts have demonstrated several pharmacological properties, such as phytoestrogenic, immunostimulant, antidiabetic, neuroprotective, hepatoprotective, anti-inflammatory, anticancer, antihyperlipidemic and gastroprotective properties [3,11,12,13,14].

Despite this growing interest, safety and toxicological assessments of date pit extracts remain limited. The available literature largely focuses on their bioactivity, mainly their antioxidant, antimicrobial, or even antimutagenic properties, without evaluating potential intrinsic toxicological risks [15]. In our previous study, we investigated the safety of ethanolic date pit extracts by screening for chemical contaminants such as mycotoxins, pesticide residues and heavy metals; only ochratoxin A (OTA) was identified in the Alig variety, and at levels below the limit of quantification (LOQ = 1.5 μg/kg) [5]. Building upon this work, the present study extends the safety assessment to evaluate the toxicological properties of the date pit extracts.

In this study, we present a preliminary safety screening of date pit ethanolic extracts from three date pit varieties (*Alig*, *Deglet Nour*, and *Kentichi*) to evaluate their mutagenic potential and in vivo toxicity. The in vivo toxicity of the three date pit varieties was evaluated using the *Galleria mellonella* model, a well-established alternative method for preliminary in vivo toxicity screening. Additionally, the Ames test, a standardized bacterial reverse mutation assay, was used for initial screening of mutagenicity using *Salmonella Typhimurium* strains TA98 and TA100 in the absence of metabolic activation. By integrating these two complementary approaches, this study provides an innovative and previously unexplored contribution to the responsible valorization of date pit by-products within the framework of a safe and sustainable circular economy.

## 2. Materials and Methods

### 2.1. Date Pits and Preparation of Extracts

Three distinct date (*Phoenix dactylifera* L.) pits—*Alig* (DA), *Deglet Nour* (DDN), and *Kentichi* (DK)—were generously provided by a Tunisian company following lyophilization. All samples were stored at −20 °C until further processing. The extracts were prepared according to the procedure previously described by Mateus et al. [5]. In brief, lyophilized pits were extracted with ethanol, a food-grade solvent [16], at a ratio of 1:10 *w*/*v*, through solid–liquid extraction (SLE), a 15 min ultrasonic bath, and a 30 min horizontal shaker at 450 rpm. The samples were then centrifuged for 10 min at 4 °C at 2862 g. A rotary evaporator (Rotavapor R-114, Büchi, Barcelona, Spain) was used to completely evaporate the ethanol (at 35 °C) after the supernatant had been filtered through Whatman^®^ No. 4 filter papers (Cytiva, Maidstone, Kent, UK). Using a spatula, the extract was taken out and stored at −20 °C, out of direct sunlight, for a maximum of six months until further processing.

### 2.2. Antimicrobial Tests

The antimicrobial potential of ethanolic date pit extracts was evaluated by determining their minimum inhibitory concentration (MIC) values against fourteen different strains of bacteria and fungi. The by-product extracts were diluted in water to obtain a concentration of 200 mg/mL. Water was chosen as the negative control since it does not affect the antibacterial activity measurement. Disk and well diffusion methods were used to measure the antibacterial activity.

#### 2.2.1. Bacterial Strains

To evaluate the antimicrobial activity of the extracts, an antimicrobial screening was performed against two Gram-positive bacterial species, i.e., *Listeria monocytogenes* (ATCC 7644) and *Staphylococcus aureus* (ATCC 25923); and three Gram-negative bacterial species, including *Campylobacter jejuni* (ATCC 33291), *Escherichia coli* (ATCC 25922), and *Pseudomonas aeruginosa* (ATCC 9027); strains of yeasts (*Candida albicans* (SCS 314), *C. parapsilosis* (ATCC 22019) and *C. glabrata* (ATCC 2001); and strains of filamentous fungi, including *Rhizopus* ssp., *Aspergillus niger*, *Penicillium expansum*, *Fusarium* spp., *Mucor racemosus* and *Alternaria* spp. All the strains were supplied from the culture collection of INIAV (Vairão, Porto, Portugal).

#### 2.2.2. Disk Diffusion Assay

Antimicrobial tests were carried out by disc-diffusion method, described by Hudzicki [17] with some modifications. For this purpose, we prepared 0.1 mL of suspension containing 108 CFU/mL of bacteria and 105 CFU/mL of yeasts. Plates with Mueller Hinton Agar (MHA) were then inoculated with S. aureus, P. aeruginosa and E. coli, while the *L. monocytogenes* was inoculated in MHA with 5% (*v*/*v*) sheep’s blood and *C. jejuni* in Columbia with 5% (*v*/*v*) horse’s blood. Discs of 9 mm in diameter were saturated with 20 μL of each extract and placed on the inoculated agar, spaced out on the surface. For the bacterial strains, disks with the antibiotic Gentamicin CN10 (10 μg/disk) and ultrapure water were used as a positive control and negative control, respectively. In case of yeast and fungi, nystatin NS100 (100 U/disc) was used as positive control. The plates were incubated at 37 °C for 24 h, except for *C. jejuni* that was incubated at 41.5 °C for 48 h, under microaerophilic conditions. The plates containing yeasts were incubated at 30 °C for 24 h, while the plates with cultures of filamentous fungi were incubated at 25 °C for 5 days. The zone of inhibition against the studied species was assessed after 24 h for bacteria and yeast and five days for fungi to assess the antimicrobial activity.

#### 2.2.3. Well Diffusion Method

The well diffusion method was based on the work of Qamar et al. [18] with minor modifications. The initial plate inoculation procedure was identical to that used in the disk diffusion method described in Section 2.2.2. Wells with a diameter of 6 mm were made in the inoculated agar, into which the extracts were deposited. The concentrated extract of each by-product was resuspended in ultrapure water at a 1:5 ratio prior to application. A volume of 20 μL of each aqueous extract was added to the wells to allow diffusion through the agar and subsequent assessment of antimicrobial activity. Wells containing only ultrapure water were used as a negative control for both bacteria and fungi. The incubation conditions and culture media were the same as those previously described for the disk diffusion method. Using a ruler, zones of inhibition were determined in millimeters and utilized as markers of antimicrobial activity. Each experiment was performed in three duplicates.

#### 2.2.4. Minimum Inhibitory Concentration (MIC)

To determine the MIC, the well diffusion method was applied. Different concentrations of the extract were prepared in dilution series from the initial extract that halved them (two-fold serial dilution), i.e., 200, 100, 50, 25 mg/mL, and so on. The MIC was determined in *S. aureus*.

### 2.3. Galleria mellonella Larvae Toxicity Assay

Following the procedure outlined by Araújo et al., [19], *G. mellonella* larvae were raised on a pollen grain diet at 25 °C in the dark and used at the end of their growth with a weight of around 250 mg. Using a microsyringe to regulate the injection volume, the larvae were injected into the haemolymph through the hindmost left proleg, which had been disinfected with 70% (*v*/*v*) ethanol beforehand.

Ten larvae were injected with 10 µL of various extract concentrations (5 MIC, 2 MIC, MIC, and 0.5 MIC) prepared in water in order to study the extracts’ in vivo toxicity. A group of larvae was injected with the same volume, but only water, as a control. The larvae were kept at 37 °C in the dark in Petri dishes. A larva was considered dead if it did not move following stimulation, and survival was observed for four days [20]. According to the scoring system created by Loh et al. [20], the *G. mellonella* health index was evaluated every 24 h using four primary parameters: activity (larvae movement), cocoon formation, melanization, and survival (Table S1). A minimum of three separate assays were used in each experiment, which was carried out in triplicate.

### 2.4. Ames Test

The Ames test was performed using a Salmonella Mutagenicity Test Kit purchased in MOLTOX^®^ (Trinova Biochem^GmbH^ -Molecular Toxicology, Inc., Boone, NC, USA), following procedures described by Maron and Ames (1983) [21] and Mortelmans and Zeiger (2000) [22] and by adapting the guidelines of OECD No. 471 [23] to 2 strains without metabolic activation [24,25]. To perform the assay, the extracts from DDN and DK were tested using water as the solvent. Four concentrations (50, 25, 12.5, and 6.25 mg/mL corresponding to 4×, 2×, 1×, and 0.5× MIC) were prepared by serial dilution. A volume of 100 µL compound per plate was applied, respecting the maximum dose of 5 mg/plate, according to the manufacturer’s guidelines.

#### 2.4.1. Bacterial Strains

*Salmonella Typhimurium* strains TA98 and TA100 were used. Strains were revived from lyophilized strains in disc format (STDiscs^T^^M^ -Molecular Toxicology, Inc., Boone, NC, USA) and cultivated overnight in nutrient broth at 37 °C with shaking to achieve 1–2 × 10^9^ CFU/mL.

To confirm the genotypes of each strain, QUAD PC^TM^(Molecular Toxicology, Inc., Boone, NC, USA) control plates containing minimal glucose agar supplemented with histidine and biotin, divided into four sectors, were inoculated using a sterile loop in a “Z” pattern with 100 µL of bacterial culture in each sector. The plates were incubated inverted at 37 °C for 48 h.

Phenotype confirmation for *S. typhimurium* strains TA98 and TA100 was carried out as follows: (a) without L-His, the strain did not grow on an agar minimum plate, validating the his-phenotype (sector I); (b) when L-His and a 10 μg crystal violet disc were present, the strain showed zonal growth inhibition on an agar minimum plate, confirming the rfa phenotype (sector II); (c) the presence of the R-factor plasmid pKM101 was confirmed by the strain’s growth on an agar minimum plate containing L-His and a 2 μg ampicillin disc (Sector III); and (d) the absence of the pAQ1 plasmid was confirmed by the strain’s inability to grow on an agar minimum plate containing L-His, 2 μg ampicillin and 1 μg tetracycline disc (Sector IV).

#### 2.4.2. Test Conditions

Serial dilutions of bacterial cultures were plated on nutrient agar to confirm cell viability and titer. Positive controls included in the kit (Control Chem™-Molecular Toxicology, Inc., Boone, NC, USA) for *S. Typhimurium* TA100 were sodium azide (1.5 µg/plate) in the absence of S9 (S9-), and for *S. Typhimurium* TA98 were daunomycin (6 µg/plate) in the absence of S9 (S9-). Negative controls consisted of 100 µL water, which is the solvent for the extracts.

For each extract, three concentrations were tested, corresponding to MIC, 2 MIC, and 4 MIC. First, two independent assays were taken as described above, with and without extract. For DDN extract, all the concentrations were re-tested a third time in order to avoid misinterpretations of the pattern evidenced by the data in the first two replicates.

#### 2.4.3. Experimental Procedure

Agar plates comprised the main medium used in the assay. Each agar plate was inoculated using the top agar method, which enables the bacteria and the tested extract to be incorporated into each plate.

In brief, 2000 μL of top agar, the histidine/biotin top agar medium, which contains biotin and a trace amount of histidine (0.05 mM each), was combined with 100 μL of each test solution, 100 μL of fresh bacterial culture (containing roughly 10^8^ viable cells), and 500 μL of sterile buffer (0.1 M phosphate buffer, pH 7.4). The buffer for the metabolic activation (S9+) assay was replaced with 500 μL of S9, which was combined with the bacteria, and test solution, as well as the top agar. A minimum agar plate was covered with a mixture of the contents from each tube. The top agar was allowed to solidify before incubation. Plates were incubated at 37 °C for 48 h. After the incubation period, the number of revertant colonies per plate was counted.

### 2.5. Statistical Analysis

Data from toxicity assays are expressed as the mean plus the standard deviation (SD) of a minimum of three separate trials, each with three replicates. Health index results were compared using ANOVA analysis with Holm–Sidak’s multiple comparisons tests with significance defined at *p* < 0.05. The log-rank Mantel–Cox statistical test was used to compute survival differences and construct Kaplan–Meier survival curves. Statistical significance was denoted as follows: *p* < 0.05 (*), *p* < 0.01 (**), *p* < 0.001 (***), and *p* < 0.0001 (****). The software GraphPad Prism^®^, version 8 (GraphPad Software, Inc., San Diego, CA, USA) was used for all tests.

## 3. Results and Discussion

### 3.1. Antimicrobial Activity

The results showed that all date pits ethanolic extracts presented antimicrobial activity against *Staphylococcus aureus*, as summarized in Table 1. The inhibition zone diameter ranged from 16.33 to 18.00 mm in disk diffusion method and 18.00 to 19.67 mm in well diffusion method, indicating consistent results between both methods.

Among the tested varieties, the *Deglet Nour* (DDN) extract showed the strongest inhibitory effect, with inhibition zones of 18.00 ± 1.00 mm (disk diffusion) and 19.67 ± 0.58 mm (well diffusion). These findings are consistent with previous reports. Bentrad et al. [26] evaluated the antimicrobial activity of *Deglet Nour* seed extracts and observed inhibition against *E. coli*, *P. aeruginosa*, *S. aureus*, and *Enterococcus faecalis*. For *S. aureus*, the inhibition zone diameter was considered to be high (15.50 ± 0.77 mm). Other studies have also demonstrated the activity of date pit extracts against *S. aureus* [27,28].

In contrast, none of the ethanolic extracts in this study exhibited inhibitory activity against *E. coli*, *C. jejuni*, *L. monocytogenes*, or *P. aeruginosa* at the concentration and volume tested. Similarly, no antifungal activity was observed against *C. albicans* or filamentous fungi. These observations align with the findings of Hussain et al. [29], who reported that extracts from seven date pit varieties (Ajwa, Fard, Khalas, Khodari, Abu Maan, Lulu, and Mabroom) exhibited no activity against *C. albicans*.

**Figure 1:**
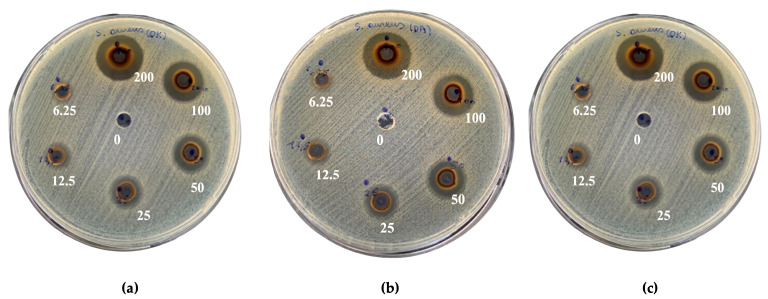
MIC assay on *S. aureus* of the aqueous extract of date seeds, with concentrations of 200, 100, 50, 25, 12.5, 6.25, and 0 mg/mL, using well diffusion assay, for (**a**) *Deglet Nour* vr.; (**b**) *Alig* vr. and (**c**) *Kentichi* vr., using 20 μL per well.

The MIC values against *S. aureus* were determined as shown in Figure 1. The MIC was determined exclusively against *S. aureus*, as it was the only microorganism against which all three extracts consistently demonstrated inhibitory activity in the diffusion assays. The MIC is the lowest extract concentration that may prevent the microorganism’s observable development. Consistent with the inhibition zone diameters, the *Deglet Nour* extract exhibited the highest antimicrobial activity, with an MIC of 6.25 mg/mL, whereas *Alig* (DA) and *Kentichi* (DK) extracts showed lower antimicrobial activity, with MIC values of 12.5 mg/mL. As expected, these MIC values were lower than the initial concentrations used in diffusion assays, reflecting the strong inhibitory effect observed at higher doses.

Overall, these results confirm that date pit ethanolic extracts have antibacterial activity, particularly against Gram-positive *S. aureus*, while showing no efficacy against Gram-negative bacteria and fungi. The stronger response of Gram-positive bacteria could be attributed to the structural differences in their cell walls, which are more permeable to phenolic compounds present in the extracts [30].

### 3.2. Toxicity Assessment of Date Pit Extracts In Vivo

To assess the in vivo toxicity of date pit ethanolic extracts, the *Galleria mellonella* larvae model was employed. *Galleria mellonella*, the larvae of the wax moth on beehives, is a well-established alternative to mammalian systems for preclinical toxicology testing [19]. The ethical acceptability, affordability, ability to be tested at mammalian body temperatures (37 °C), and functional resemblance to the innate immune systems of mammals are some of the benefits of this invertebrate model [31]. Additionally, there is a favorable correlation between *G. mellonella* results and other invertebrate models as well as mammalian models [20,32].

**Figure 2:**
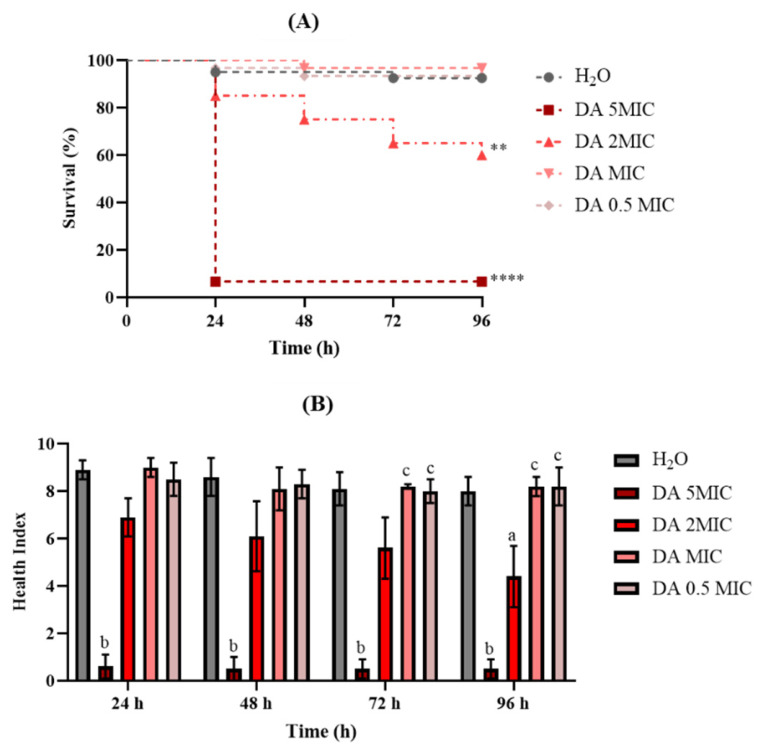
Toxicity evaluation of date pit extract form *Alig* variety (DA) in the *G. mellonella* model, with (**A**) survival curves of larvae and (**B**) health index of larvae, both over 96 h. ** Significant differences between negative control and 2 MIC concentration (*p* < 0.01). **** Significant differences between negative control and 5 MIC (*p* < 0.0001). ^a^ Significant difference between 24 h and other time points for each concentration (*p* < 0.05); ^b^ significant difference between 5 MIC and other concentrations, for each time (*p* < 0.05); ^c^ significant difference between 2 MIC and MIC/0.5 MIC, for each time point (*p* < 0.05).

**Figure 3:**
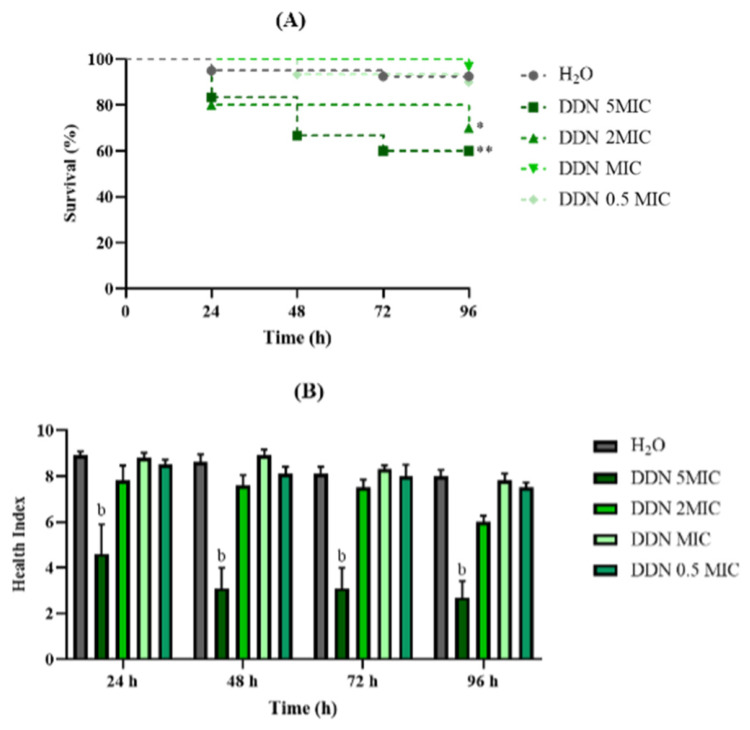
Toxicity evaluation of date pit extract form *Deglet Nour* variety (DDN) in the *G. mellonella* model, with (**A**) Survival curves of larvae and (**B**) health index of larvae, both over 96 h. * Significant differences between negative control and 2 MIC (*p* < 0.05). ** Significant differences between negative control and 5 MIC (*p* < 0.01). ^b^ Significant difference between 5 MIC and other concentrations, for each time (*p* < 0.05).

**Figure 4:**
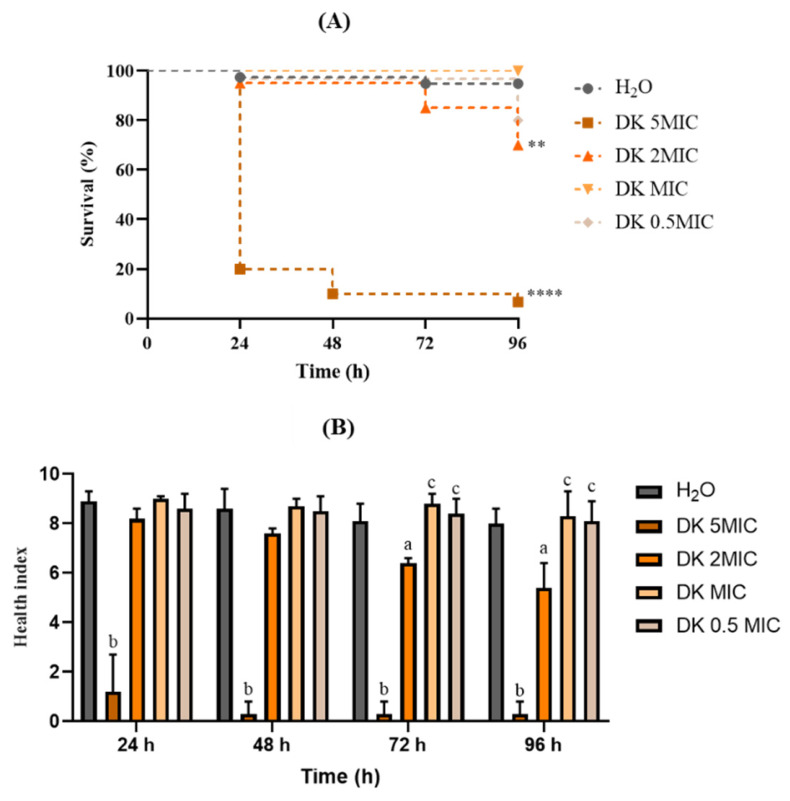
Toxicity evaluation of date pit extract form *Kentichy* variety (DK) in the *G. mellonella* model, with (**A**) Survival curves of larvae and (**B**) health index of larvae, both over 96 h. ** Significant differences between negative control and 2 MIC (*p* < 0.01). **** Significant differences between negative control and 5 MIC (*p* < 0.0001). ^a^ Significant difference between 24 h and other time points for each concentration (*p* < 0.05); ^b^ significant difference between 5 MIC and other concentrations, for each time *(p* < 0.05); ^c^ significant difference between 2 MIC and MIC/0.5 MIC for each time point (*p* < 0.05).

To assess the toxicity of the ethanolic date pit extracts, the survival rate and health index of *G. mellonella* were determined [20]. Four different concentrations of each extract (5 MIC, 2 MIC, MIC, and 0.5 MIC) were injected into *G. mellonella* larvae, and the health index was evaluated for 96 h, as shown in Figure 2, Figure 3 and Figure 4.

At the highest concentration (5 MIC), all extracts were toxic to the larvae. For DA and DK extracts, almost all larvae were dead after 24 h (*p* < 0.0001). A MIC of 2 was less toxic to the larvae, although by 96 h only approximately 60% of the larvae were alive in all extracts (*p* < 0.01). In addition, the health index was significantly lower than those of the water control for DA and DK at 96 h and 72 h, respectively (*p* < 0.05). In contrast, for DDN extract, the health index at 96 h did not differ significantly from the control (*p* > 0.05). Concentrations at or below MIC did not show toxic effects on *G. mellonella* larvae in any of the extracts, with both survival rates and health indices comparable to the control group (*p* > 0.05).

To the best of our knowledge, this is the only study to use this *G. mellonella* model to evaluate the toxicity of date pit ethanolic extracts. Previous applications of this invertebrate system have focused on other natural substances, such as propolis [33], demonstrating its suitability for evaluating complex plant-derived materials.

Most of the toxicity assays in date pit extracts were performed using other in vivo models for both acute and chronic toxicity [4]. For example, Mohamed Doha A. and Al-Okbi [34] assessed the acute oral toxicity of date seed extract in male and female albino mice. The median lethal dose (LD_50_) was 6.75 g/kg b.w., which is 52.4 g for an adult weighing 70 kg. At a dosage of 4 g/kg b.w., all test animals survived, and no side effects were noted, indicating a NOAEL (No Observed Adverse Effect Level) at that concentration. Moreover, Ali Zarie et al., [35] investigated the chronic effects of daily administration of date seed extracts from several cultivars (Qatara, Barhi, Ruthana) to Wistar rats for 28 days at doses of 300 and 600 mg/kg b.w., reporting no adverse effects.

The findings from the *G. mellonella* model are in line with these mammalian studies, supporting the general low acute toxicity of date pit ethanolic extracts at moderate concentrations. While the observed larval mortality at high extract doses highlights the importance of dose-dependent evaluation, the mechanistic basis of this toxicity was not investigated in the present study. Future work incorporating biochemical and histopathological analyses of the *G. mellonella* model would substantially strengthen the toxicological interpretation of these high-dose effects. Nevertheless, as a preliminary invertebrate model, *G. mellonella* does not replicate mammalian digestion or metabolism; further studies, including mammalian cytotoxicity assays and oral toxicity evaluation, are required before definitive conclusions regarding safety can be established.

### 3.3. Mutagenicity of Date Pits Extracts

While in vivo models such as *Galleria mellonella* provide valuable information on systemic toxicity, the evaluation of genotoxic potential requires specific assays targeting DNA damage. Therefore, the mutagenic potential of the extracts was assessed using the Ames test.

The raw data from the Ames test on the two ethanolic date pit extracts is shown in Tables S2–S5. DDN was selected for being apparently safer at higher concentrations; while DK presents high toxicity at high concentration (90% mortality at 5 MIC). Each table reports the number of revertants per plate, for *S. Typhimurium* strains TA98 and TA100, treatment with extracts, and in the absence (S9-) of metabolic activation. The summarized results are shown in Table 2, which compares the mutagenic indexes of the ethanolic extracts from *Deglet Nour* (DDN) and *Kentichi* (DK) date pit varieties with those of known genotoxic positive controls. The ratio of the mean number of revertants per plate observed for each test extract to that of the corresponding negative (solvent) control is known as the mutagenic index (MI) [21].

According to the criteria described by Ames et al., (1975) [36], a substance can be considered mutagenic when it induces at least a twofold increase in revertant colonies compared to the negative control in at least one tested concentration. As shown in Table 2, under all applied concentrations of date pit extract (6.25 to 25 mg/mL), MI values remained below 2 for both extracts and both strains, except for DDN on TA98, where MI values exceeded 2 at all tested concentrations (3.03, 3.03, and 2.78 at MIC, 2MIC, and 4MIC, respectively). Although the absence of a clear dose–response relationship and the normal MI values obtained with TA100 reduce the probability of a genuine mutagenic effect, the consistently elevated MI on TA98, combined with the high variability and the anomalous negative control counts (discussed below), means that mutagenic potential for DDN on TA98 cannot be conclusively excluded under the current experimental conditions. Moreover, the relatively high standard deviations observed across multiple conditions in Table 2, particularly for DDN on TA98 (e.g., SD of ±2.17 at MIC), further challenges confidence in the precision of the MI estimates and highlights the importance of future assessment. Confirmation in a follow-up study using freshly validated strains and a fully compliant OECD 471 protocol would be relevant. However, it is possible to state that under the conditions of this preliminary screening, and considering the data collectively, the extracts did not exhibit a clear mutagenic profile.

Comparable observations were reported by Shoji et al. [37], who evaluated the mutagenicity of unripe apple extracts. A slight increase in revertants was observed in the TA98 strain at 2.5 mg/plate without S9 activation, while no significant changes occurred in other strains (TA100, TA1535, TA1537, *E. coli* WP2) at concentrations up to 5.0 mg/plate. These findings were likewise interpreted as non-mutagenic.

In the present work, ethanolic extracts of date pits were tested, whereas several earlier studies used aqueous extracts [38] or extracts prepared from date pulp [39,40]. Given that more than 3000 date palm varieties have been identified worldwide [41], differences in genotype and growth conditions may induce variability in biological effects. Studies on other cultivars such as Ajwa [10,39,42], Barheewas [43] and Khalas [40,44] have reported antimutagenic or anticancer properties, highlighting the influence of variety on bioactivity.

Some limitations of this study should be acknowledged. First, metabolic activation was not included. Many carcinogens are pro-mutagens that require biotransformation through cytochrome P450–dependent metabolism, which occurs mainly in the mammalian liver but not in *S. Typhimurium* [45]. The absence of S9 activation therefore means that the scope of the mutagenicity conclusions is strictly limited to direct-acting mutagens, and that a negative result under these conditions cannot be extrapolated to a negative result under full OECD 471-compliant conditions.

Second, and critically, considering the spontaneous revertant counts for negative controls were below the generally acceptable historical range (20–50 for T98 strain, and 75–200 for T100 strain), it may indicate partial loss of genetic integrity in the test strain [22]. However, the positive controls performed as expected in all assays (>500 colo-nies/plate for TA98; >350 for TA100), confirming that the strains retained their capacity for reversion under inducing conditions and that the assay remained functional.

Third, only two bacterial strains were tested. The OECD Test Guideline No.471 [23] recommends that at least five strains should be used to cover different mutation mechanisms. The *S. Typhimurium* strains used in this Ames test present different DNA sequence specificity, TA98 detects frameshifts mutation (additions or deletions of one or more bases), while TA100 detects base pair substitution (substitution of a leucine (GAG/CTC) by a proline (GGG/CCC), both on GC base pairs. The percentage of mutagens identified by these two strains includes over 90% of the cases [46]. Other *S. Typhimurium* strains could be used, such as TA102 or TA104 strains or even *E. coli* WP2 strains which have an AT base pair at the primary reversion site; these would provide broader coverage of mutagenic mechanisms, which is required for a complete assessment [22,46]. Taken together, these limitations mean that the Ames test data presented here should be regarded strictly as a preliminary screening. The results do not support a definitive conclusion of non-mutagenicity and must be confirmed in a follow-up study conducted under conditions that fully comply with OECD Guideline 471.

Most of the report studies using date fruit or date pit extracts focused on antimutagenic rather than mutagenic properties, using known mutagens; for example, benzo(a)pyrene or N-Nitroso-N-methylurea (NMU). Thus, they do not assess their safety through the Ames test, but their potential therapeutic effects [38,39,40,41,42,43,44,45,46,47,48]. Other authors have investigated the activity of isolated polyphenols, e.g., gallic acid extracted and isolated from date pits [10], rather than the whole extract. However, whole extracts are complex mixtures where synergistic or antagonistic interactions among components can significantly influence biological responses. Moreover, a number of factors, including cultivar, location, soil and climate, harvest ripeness, and extraction technique, affect the chemical composition of agri-food by-products, including date pits [5]. For instance, establishing a correlation between the phenolic profile of each variety and the differing mutagenicity responses observed, particularly the high MI values for DDN in TA98 compared with DK, would help to clarify whether specific compounds or synergistic interactions are responsible for this variety-dependent effect. Consequently, the mutagenic potential must be evaluated for each new extract intended for food, nutraceutical, or packaging applications.

## 4. Conclusions

This study provides a first preliminary safety screening of ethanolic extracts obtained from date pits of the *Alig*, *Deglet Nour*, and *Kentichi* varieties. Under the conditions tested, the results indicate that extracts do not exhibit mutagenic or toxic effects at concentrations at or below the MIC, although variety-dependent differences were observed at higher concentrations.

This preliminary safety screening strategy, combining in vitro (Ames test) and in vivo (*G. mellonella*) approaches, represents a useful and ethical first-line screening for the preliminary safety evaluation of natural extracts intended for functional ingredients or active agents in food and food packaging within a sustainable circular economy.

Nevertheless, these findings are preliminary, and further studies are required before definitive safety conclusions can be drawn, including S9 metabolic activation and a broader panel of bacterial strains, as recommended by OECD Guideline 471, as well as complementary mammalian cytotoxicity and oral toxicity assays.

## Figures and Tables

**Table 1:** Results of the disk diffusion method for determining antimicrobial activity against bacteria expressed as inhibition zone diameter (mm).

	Date Seeds			Positive Control
Microorganism	DA	DDN	*DK*	
Bacteria				*CN 10*
*E. coli*	n a	na	na	25.00 ± 0.10
*P. aeruginosa*	na	na	na	23.33 ± 0.58
*C. jejuni*	na	na	na	37.67 ± 0.58
*L. monocytogenes*	na	na	na	31.00 ± 1.00
*S. aureus*	dd 16.77 ± 1.15 wd 18.33 ± 0.58	dd 18.00 ± 1.00 wd 19.67 ± 0.58	dd 16.33 ± 1.15 wd 18.00 ± 0.58	dd 26.00 ±1.00 na
Yeasts				*NS 100*
*C. albicans*	na	na	na	25.00 ± 1.00
*C. parapsilosis*	na	na	na	28.00 ± 1.00
*C. glabrata*	na	na	na	28.33 ± 0.58
Fungi				*NS 100*
*Rhizopus* ssp.	na	na	na	9.00 ± 0.10
*A. niger*	na	na	na	25.00 ± 1.0
*P. expansum*	na	na	na	15.33 ± 0.58
*Fusarium* spp.	na	na	na	7.67 ± 0.58
*M. racemosus*	na	na	na	19.33 ± 0.58
*Alternaria* spp.	na	na	na	16.33 ± 0.58

na—not applicable; dd—disk diffusion method; wd—well diffusion method.

**Table 2:** Mean revertants per plate and mutagenic index of date pit extract from *Deglet Nour* (DDN) and *Kentichi* (DK) varieties, without metabolic activation (S9-), measured in *S. Typhimurium* TA98 and TA100 bacterial strains, treated with extracts at various doses.

	DDN		DK	
Revertants	TA98	T100	TA98	T100
Control (−)	9.17 ± 6.85	45.00 ± 44.40	16.75 ± 6.29	42.25 ± 3.77
MIC	19.50 ± 7.82	43.33 ± 46.89	13.00 ± 7.87	40.25 ± 11.7
2MIC	23.50 ± 10.07	60.50 ± 48.03	18.75 ± 5.32	21.5 ± 16.26
4MIC	23.67 ± 13.54	49.00 ± 41.44	8.00 ± 6.38	24.25 ± 24.72
Control (+)	>500/plate	>350/plate	>500/plate	>350/plate
Mutagenic Index (MI)				
MIC	3.03 ± 2.17	0.81 ± 0.21	0.73 ± 0.23	0.96 ± 0.35
2MIC	3.03 ± 1.17	1.47 ± 0.57	1.15 ± 0.15	0.52 ± 0.13
4MIC	2.78 ± 0.82	1.98 ± 1.58	0.42 ± 0.28	0.59 ± 0.74

Results expressed by mean ± SD (for at least two independent assays, with 2 replicates); MIC = 6.25 mg/mL. Negative controls consisted of water. Positive controls consisted of *S. Typhimurium* TA100 NaN3 (-S9) and for *S. Typhimurium* TA98 daunomycin (-S9).

## Data Availability

The original contributions presented in the study are included in the article/Supplementary Materials. Further inquiries can be directed to the corresponding authors.

## Supplementary Materials

The following supporting information can be downloaded at https://www.mdpi.com/article/10.3390/foods15122168/s1, (Table S1: Health index scoring system, according to Loh et al. (2013); Table S2: Number of revertants/plate for *S. typhimurium* TA98 strain, treated with date pit extract from *Deglet Nour* variety (DDN); Table S3: Number of revertants/plate for *S. typhimurium* TA100 strain, treated with date pit extract from *Deglet Nour* variety (DDN); Table S4: Number of revertants/plate for *S. typhimurium* TA98 strain, treated with date pit extract from *Kentichy* variety (DK); Table S5: Number of revertants/plate for *S. typhimurium* TA100 strain, treated with date pit extract from *Kentichy* variety (DK).

## Abbreviations

The following abbreviations are used in this manuscript:

ATCC	Multidisciplinary Digital Publishing Institute
CFU	Colony-Forming Units
DA	Ethanolic extract from date pit of the *Alig* variety
DDN	Ethanolic extract from date pit of the *Deglet Nour* variety
DK	Ethanolic extract from date pit of the *Kentichi* variety
DPPH	2,2-Diphenyl-1-picrylhydrazyl assay
L-His	L-Histidine
LOQ	Limit of Quantification
MIC	Minimum Inhibitory Concentration
OTA	Ochratoxin A
pKM101	Plasmid carried by certain *S. Typhimurium*
TA100	*Salmonella Typhimurium* strain used in Ames test to detect base pair substitution mutations
TA98	*Salmonella Typhimurium* strain used in Ames test to detect frameshift mutations
TFC	Total Flavonoid Content
TPC	Total Phenolic Content
UHPLC-ToF-MS	Ultra-High-Performance Liquid Chromatography coupled with Time-of-Flight Mass Spectrometry
