# Supplementary material for: Preliminary Assessment of Mutagenicity and In Vivo Toxicity of Date Pit Ethanolic Extracts: Safety Screening for Circular Economy Applications
Source: Foods. 2026 Jun 16;15(12):2168. doi: 10.3390/foods15122168 (PMC13297971; doi:10.3390/foods15122168)
Supplement: Supplementary file 1 [file foods-15-02168-s001.zip › foods-4324570-supplementary.pdf]

Supplementary Material

Mutagenicity and *In Vivo* Toxicity of Date Pit Ethanolic Extracts: Safety Assessment for Circular Economy Applications

Ana Rita Soares Mateus [1,2,3,4], João Vindeirinho [1,5,6], Khaoula Khwaldia [7], Joana Castro [1,8], Daniela Araújo [1], Angelina Pena [2,3], Matheus Lemos [1], Ana Rita Barata [1,4], Maria José Saavedra [9], Gonçalo Almeida [1,4,10], Ana Sanches Silva [2,4,10,*], Carina Almeida [1,5,6,8,*]

Table S1. Health index scoring system, according to Loh et al. (2013)

Category	Description	Score
**Activity**	No activity	0
	Minimal activity on stimulation	1
	Active when stimulated	2
	Active without stimulation	3
**Coccon formation**	No cocoon	0
	Partial cocoon	0.5
	Full cocoon	1
**Melanization**	Complete melanization (black)	0
	Dark spots on brown wax worm	0
	≥ 3 spots on beige wax worm	2
	< 3 spots on beige wax worm	3
	No melanization	4
**Survival**	Dead	0
	Alive	2

Reference:

Loh, J.M.; Adenwalla, N.; Wiles, S.; Proft, T. *Galleria Mellonella* Larvae as an Infection Model for Group A Streptococcus. *Virulence* **2013**, 4, 419–428, doi:10.4161/viru.24930.

Table S2. Number of revertants/plate for *S. typhimurium* TA98 strain, treated with date pit extract from *Deglet Nour* variety (DDN).

	Replicate 1				Replicate2				Replicate 3			
	#1	#2	Mean	SD	#1	#2	Mean	SD	#1	#2	Mean	SD
Control (-)	14	21	17.50	4.95	5	4	4.5	0.71	6	5	5.5	4.95
MIC	19	14	16.50	3.54	12	14	13	1.41	27	31	29	3.54
2MIC	32	36	34.00	2.83	16	10	13	4.24	19	28	23.5	2.83
4MIC	42	38	40.00	2.83	12	9	10.5	2.12	22	19	20.5	2.83
Control (+)	More than 500/plate				More than 500/plate				More than 500/plate			

Table S3. Number of revertants/plate for *S. typhimurium* TA100 strain, treated with date pit extract from *Deglet Nour* variety (DDN).

	Replicate 1				Replicate2				Replicate 3			
	#1	#2	Mean	SD	#1	#2	Mean	SD	#1	#2	Mean	SD
Control (-)	102	97	99.50	3.54	34	32	33.00	1.41	2	3	2.50	0.71
MIC	101	103	102.00	1.41	22	31	26.50	6.36	2	1	1.50	0.71
2MIC	107	110	108.50	2.12	62	78	70.00	11.31	4	2	3.00	1.41
4MIC	100	100	100.00	0.00	37	38	37.50	0.71	9	10	9.50	0.71
Control (+)	More than 350/plate				More than 350/plate				More than 350/plate			

Table S4. Number of revertants/plate for *S. typhimurium* TA98 strain, treated with date pit extract from *Kentichy* variety (DK).

	Replicate 1				Replicate2			
	#1	#2	Mean	SD	#1	#2	Mean	SD
Control (-)	11	12	11.50	0.71	20	24	22.00	2.83
MIC	8	5	6.50	2.12	17	22	19.50	3.54
2MIC	17	12	14.50	3.54	23	23	23.00	0.00
4MIC	2	3	2.50	0.71	14	13	13.50	0.71
Control (+)	More than 500/plate				More than 500/plate			

Table S5. Number of revertants/plate for *S. typhimurium* TA100 strain, treated with date pit extract from *Kentichy* variety (DK).

	Replicate 1				Replicate2			
	#1	#2	Mean	SD	#1	#2	Mean	SD
Control (-)	42	45	43.50	2.12	45	37	41.00	5.66
MIC	29	33	31.00	2.83	55	44	49.50	7.78
2MIC	9	6	7.50	2.12	37	34	35.50	2.12
4MIC	4	2	3.00	1.41	42	49	45.50	4.95
Control (+)	More than 350/plate				More than 350/plate			
